# Large language models open new way of AI-assisted molecule design for chemists

**DOI:** 10.1186/s13321-025-00984-8

**Published:** 2025-03-24

**Authors:** Shoichi Ishida, Tomohiro Sato, Teruki Honma, Kei Terayama

**Affiliations:** 1 Graduate School of Medical Life Science, Yokohama City University, 1-7-29, Suehiro-cho, Tsurumi-ku, Yokohama, Kanagawa 230-0045 Japan; 2 MolNavi LLC, #402 Wizard building 1-4-3 Sengen-cho Nishi-ku, Yokohama, Kanagawa 220-0072 Japan; 3 RIKEN Center for Integrative Medical Sciences, 1-7-22 Suehiro-cho, Tsurumi-ku, Yokohama, 230-0045 Japan; 4 RIKEN Center for Advanced Intelligence Project, 1-4-1, Nihonbashi, Chuo-ku, Tokyo, 103-0027 Japan; 5 MDX Research Center for Element Strategy, Institute of Science Tokyo, 4259, Nagatsuta-cho, Midori-ku, Yokohama, Kanagawa 226-8501 Japan

## Abstract

Recent advancements in artificial intelligence (AI)-based molecular design methodologies have offered synthetic chemists new ways to design functional molecules with their desired properties. While various AI-based molecule generators have significantly advanced toward practical applications, their effective use still requires specialized knowledge and skills concerning AI techniques. Here, we develop a large language model (LLM)-powered chatbot, ChatChemTS, that assists users in designing new molecules using an AI-based molecule generator through only chat interactions, including automated construction of reward functions for the specified properties. Our study showcases the utility of ChatChemTS through de novo design cases involving chromophores and anticancer drugs (epidermal growth factor receptor inhibitors), exemplifying single- and multiobjective molecule optimization scenarios, respectively. ChatChemTS is provided as an open-source package on GitHub at https://github.com/molecule-generator-collection/ChatChemTS.

**Scientific contribution**

ChatChemTS is an open-source application that assists users in utilizing an AI-based molecule generator, ChemTSv2, solely through chat interactions. This study demonstrates that LLMs possess the potential to utilize advanced software, such as AI-based molecular generators, which require specialized knowledge and technical skills.

**Supplementary Information:**

The online version contains supplementary material available at 10.1186/s13321-025-00984-8.

## Introduction

Artificial intelligence (AI)-based techniques for molecular designs are becoming promising methods for designing synthetically accessible and insightful molecules with desired functionalities [1–8]. Research articles on these techniques have been reported in a wide range of fields, from material design to drug discovery. In terms of material design, fluorescent [4] and photofunctional [1, 5] molecules have been designed using AI-based molecule generators, and the designed molecules were successfully experimentally validated to exhibit the desired properties. Similarly, in drug discovery, new proton pump inhibitors [6] and inhibitors for targeting antifibrotic effects [7] were designed and demonstrated their good inhibitory effects. The AI-based molecule generators used in the above studies represent just a fraction of the techniques that have been developed thus far [9–25], and applying and testing various promising molecule generators to solve real-world problems is vital for achieving further advancements.

While various AI-based molecule generators have made significant progress toward practical applications, their effective utilization still requires specialized knowledge and skills concerning AI techniques [26]. This high level of expertise presents a critical obstacle to the widespread adoption of AI-based molecule generators. The effective use of these methods necessitates a deep understanding of how to design reward functions that appropriately represent the desired functionalities and the ability to configure the set conditions according to the specifications of each AI-based molecule generator. In chemical, pharmaceutical, and other industries, the complexity of utilizing AI-based molecule generators and the need for skills such as machine learning (ML,) to design reward functions pose significant obstacles that prevent users from easily adopting these technologies for their projects. These challenges complicate the effective utilization of AI-based molecule generators to solve real-world problems, especially for researchers and developers who possess expert knowledge and skills in chemistry but are not well versed in AI techniques.

To address these challenges, we developed ChatChemTS, a large language model (LLM)-powered chatbot that assists users in utilizing ChemTSv2 [11]—AI-based molecule generator with experimental validations for various molecule designs [1, 3–5]—through only interactive chats. All users are merely required to express a request to ChatChemTS via chat, and ChatChemTS then prepares the appropriate reward functions, configures the desired conditions, and executes ChemTSv2 for the users. In addition, ChatChemTS provides a tool for analyzing the output molecule generation results. ChatChemTS is based on a ReAct framework so that it can address the whole workflow of general AI-based molecule generators, and the framework employs the generative pretrained transformer (GPT) model of OpenAI, which has shown the great potential as an LLM chemistry agent to perform chemistry-related tasks [27–33]. As example applications of ChatChemTS, we performed two de novo molecular design tasks, one involving a photofunctional organic molecule and another concerning a kinase inhibitor, as single- and multiobjective molecule optimization problems, respectively. Notably, users only need to prepare data related to the physicochemical properties of molecules or information about the target proteins of interest to perform AI-based molecule designs with ChatChemTS. We show that this concept of utilizing an LLM as an assistant of AI-based molecule generators can be easily introduced to various generators developed with organized application structures, such as ChemTSv2. This study also showed the potential of LLMs not only to use software that requires simple inputs, such as a SMILES string, and advanced APIs for operating robots as tools, as shown in previous studies [27, 29], but also to be able to utilize highly flexible software, AI-based molecular generators, as tools. The ChatChemTS application is publicly available on GitHub at https://github.com/molecule-generator-collection/ChatChemTS.

## Results

### Implementation of ChatChemTS

**Fig. 1:**
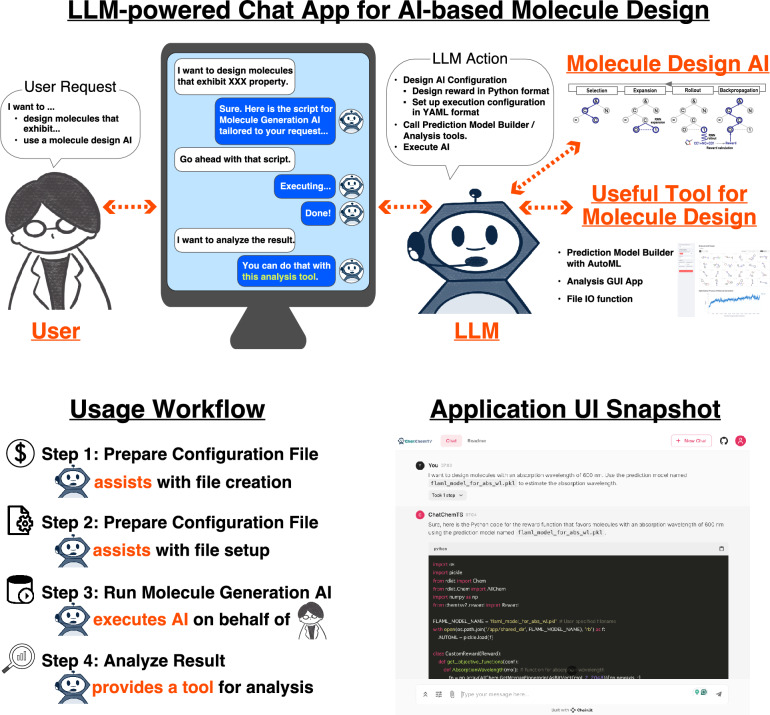
Overview of ChatChemTS. The visual workflow of ChatChemTS is shown in the upper panel. A user can utilize ChatChemTS via chat in a web browser on a local laptop, and ChatChemTS assists users in designing molecules through AI. The schematic usage workflow of ChatChemTS is shown in the lower left panel. A snapshot of the user interface (UI) of this application is shown in the lower right panel. The UI was built with Chainlit and provides the users with an intuitive chat experience

ChatChemTS was developed based on LLMs to help users employ ChemTSv2 through interactive chats, as shown in Fig. 1.

**Fig. 2:**
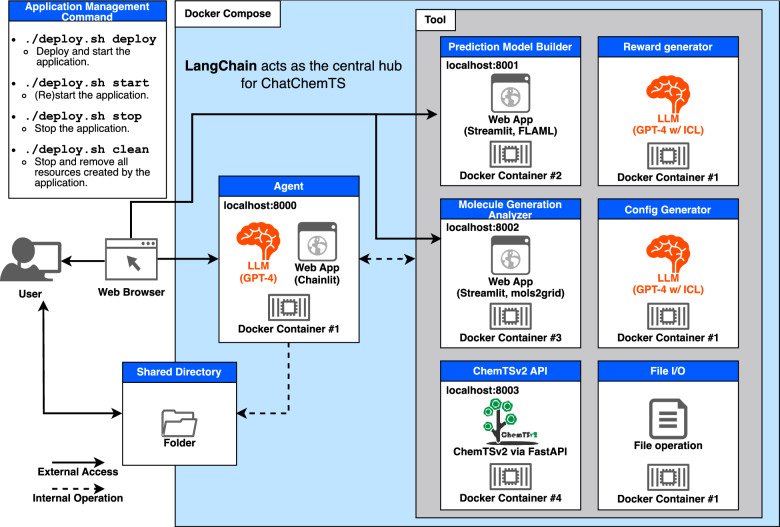
System architecture of ChatChemTS. LangChain was used to implement the ReAct framework in ChatChemTS. LLMs were used in the agent, the reward generator tool, and the configuration generator tool. Additionally, both tools utilized LLMs specialized in the corresponding tasks using in-context learning (ICL). ChatChemTS was containerized using Docker, allowing for easy deployment to user environments through Docker Compose. Users can employ ChatChemTS via local web browsers, such as Google Chrome

The ChatChemTS application employs a ReAct framework [34] that enables LLMs to generate reasonable responses and take appropriate actions, including the use of predefined tools, as shown in Fig. 2. The predefined tools in ChatChemTS include a reward generator, a prediction model builder, a configuration generator, a ChemTSv2 application programming interface (API), a molecule generation analyzer, and a file writing tool. In this study, the configuration included molecule generation parameters in ChemTSv2, such as molecular filtering functions and an exploration parameter *c* [11]. ChemTSv2 provides common filtering criteria, including the synthetic accessibility score (SAScore) [35] and Lipinski’s rule of five [36]. The parameter c balances exploration and exploitation in the upper confidence bound (UCB1) score. In molecule generation, a higher c value (e.g., 1.0) tends to generate molecules with diverse scaffolds, while a lower c value (e.g., 0.1) tends to focus the search on optimizing molecules that appear promising during exploration. To offer high-quality responses, multiple LLMs were utilized in ChatChemTS and specifically tailored for distinct roles, such as facilitating user interactions and crafting reward function designs; thus, the use of verbose and ambiguous prompts that may elicit irrelevant responses was minimized in each LLM.

The platform was developed primarily by incorporating LangChain [37], GPT-4 (gpt-4-0613) from OpenAI [38], and FastAPI [39] for backend operations, as well as Streamlit [40] and Chainlit [41] for frontend implementations, as shown in Fig. 2. To enhance the scalability of the application, the computing environments assigned to each role were containerized using Docker, ensuring that no interference occurred among them. Docker Compose [42] was utilized to implement a streamlined deployment process leveraging multiple Docker images, facilitating the automatic deployment of the application in a Docker-compatible environment. A demonstration movie of the workflow for ChatChemTS is shown in Supplementary Movie 1.

### Example applications of ChatChemTS

**Fig. 3:**
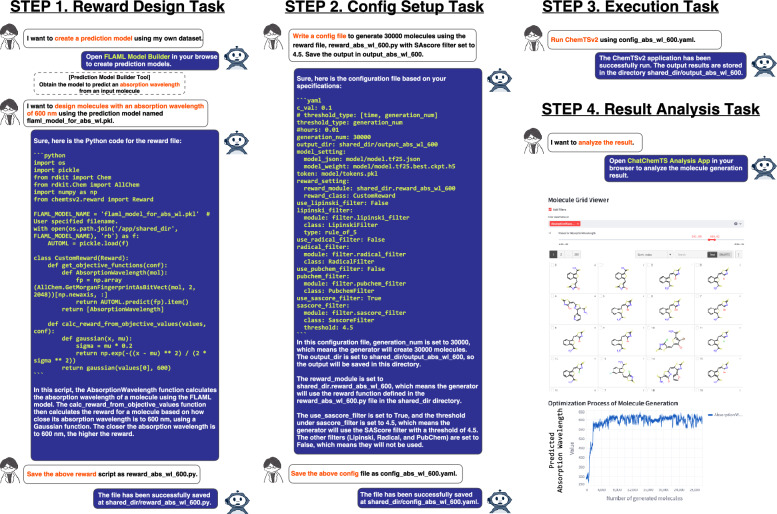
Application of ChatChemTS in designing chromophore. This demonstration aimed to design molecules with absorption wavelengths of 600 nm. In step 1, based on the observed user requests, ChatChemTS created a prediction model to predict the absorption wavelength from an input molecule. The training dataset was prepared using 50000 compounds with DFT-calculated absorption wavelengths at the B3LYP/6-31 G* level [44] via the prediction model builder. The prediction model, saved with the name flaml_model_for_abs_wl.pkl during its construction using the prediction model builder, was used in the reward function. In step 2, ChatChemTS set up the configuration of ChemTSv2 according to the specifications provided by the user. In step 3, ChatChemTS executed the ChemTSv2 using the prepared configuration file. In step 4, a user analyzed the molecule generation results. The right panel shows examples of molecules with absorption wavelengths of approximately 600 nm and the optimization process of the molecule generation task. Expanded views of each column are provided in the Fig. S2

**Fig. 4:**
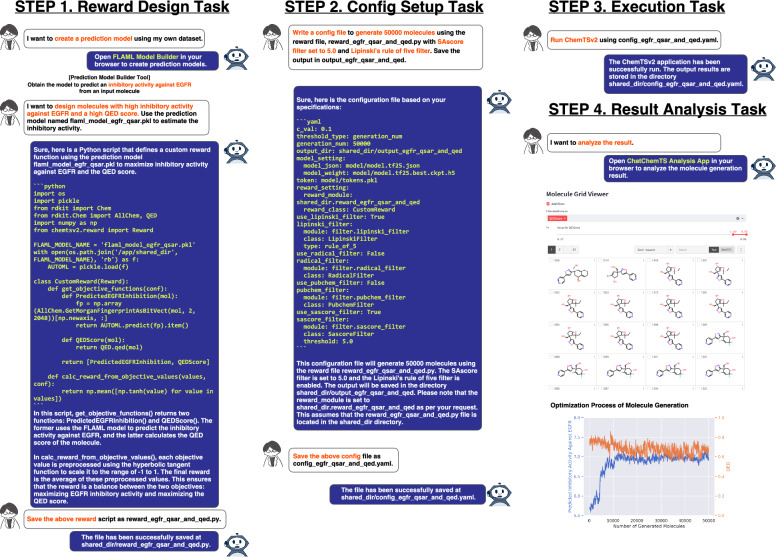
Application of ChatChemTS in designing EGFR inhibitors. This demonstration aimed to design molecules that exhibited inhibitory activities against EGFR and high QED scores. In step 1, based on the observed user requests, ChatChemTS created a prediction model to predict the inhibitory activity of an input molecule. To prepare the training dataset, compounds that possessed activity data for the protein associated with UniProt ID P00533 were retrieved from the ChEMBL database via the prediction model builder, and this task was followed by preprocessing. The prediction model was used in the reward function. The prediction model, saved with the name flaml_model_egfr_qsar.pkl during its construction using the prediction model builder, was used in the reward function. In step 2, ChatChemTS set up the configuration of ChemTSv2 according to the specifications provided by the user. In step 3, ChatChemTS executed the ChemTSv2 using the prepared configuration file. In step 4, a user analyzed the molecule generation results. The right panel shows the generated molecules ranked in descending order of their reward scores, provided that the QED scores were 0.89 or higher and the optimization process of the molecule generation. Expanded views of each column are provided in the Fig. S4

To demonstrate the utility of ChatChemTS, we performed de novo designs of a chromophore and an epidermal growth factor receptor (EGFR) inhibitor, the latter of which is a common treatment for breast and lung cancers [43]. These designs illustrated examples of single- and multiobjective molecule optimization tasks, respectively. The aim of the chromophore design process was to optimize molecules so that they possessed a specific absorption wavelength. On the other hand, the EGFR inhibitor design process focused on obtaining molecules with high inhibitory activity and high drug likeness scores. All file names presented in Fig. 3 and Fig. 4 are arbitrary and can be freely modified by the user when using ChatChemTS. Detailed explanations of both demonstrations are described below.

#### Chromophore design

Figure 3 shows a chat-based demonstration of the task of designing chromophores using ChatChemTS.

The enlarged versions of each column in the figure are provided in Fig. S2. All processes described hereafter were successfully carried out through chat interactions and operations in graphical user interface (GUI) applications: prediction model builder and analysis tools. The prediction model builder of ChatChemTS provides a function for constructing ML models that predict a molecule property when provided a dataset in comma-separated values (CSV) format (see the Methods section for details). First, the prediction model builder tool was used to create an ML model for predicting an absorption wavelength from an input molecule. The training dataset was prepared in CSV format and included 50000 molecules with absorption wavelengths calculated using density functional theory (DFT) at the B3LYP/6-31 G* level [44]. The AutoML parameters were set with a test dataset ratio of 0.1 and a budget time of one hour; the estimators list and metrics were automatically selected by the Fast Library for Automated Machine Learning and Tuning (FLAML) during the AutoML search process. The best model was the light gradient boosting machine (LightGBM), and its correlation coefficient for the test dataset was 0.93. The above GUI-based operations and their results are shown in Fig. S1, and the demonstration movie of the prediction model builder can be seen in Supplementary Movie 2. Next, a reward function and a configuration were designed via chat based on the following conditions: the target absorption wavelength was set to 600 nm, the exploration parameter *c* was set to 0.1, the number of generated molecules was set to 30000, and an SAscore filter with a threshold of 4.5 was used. Then, ChemTSv2 was executed via chat using the above reward function and configuration files. Finally, the analysis tool was utilized to analyze the molecule generation results. As shown in the optimization process of Fig. 3 (right panel), ChatChemTS successfully designed molecules with predicted absorption wavelength of approximately 600 nm.

#### EGFR inhibitor design

Figure 4 shows a chat-based demonstration of the task of designing EGFR inhibitors using ChatChemTS.

The enlarged versions of each column in the figure are provided in Fig. S4. The subsequent processes were all accomplished through chat interactions and operations in the GUI applications. The prediction model builder of ChatChemTS provides a function for constructing regression ML models that predict inhibitory activity against a target protein by simply specifying its Universal Protein Resource ID (UniProt ID; see the Methods section for details). In this demonstration, UniProt ID P00533 was used as the input of the prediction model builder tool to retrieve molecules with inhibitory activities against EGFR from the ChEMBL database. ML models were designed to take a molecule as input and predict a pChEMBL value, representing the negative base-10 logarithm of the half-maximal response concentration, potency, and affinity. The retrieved dataset was preprocessed as follows: (1) deduplicating molecules by leaving the maximum pChEMBL values; (2) retaining records by assay type of Binding; (3) filtering out records with assay descriptions, which contained the mutat, covalent, and irreversible substrings; and (4) removing the activity types of the half-maximal effective concentration (EC50) and half-maximal active concentration (AC50). Consequently, the dataset size was 7141 compounds, and the dataset was used in the training step. The AutoML parameters were set with a test dataset ratio of 0.1 and a budget time of one hour; the estimator list and metrics were automatically selected by the FLAML during the AutoML search process. The option to standardize the objective variable was applied to facilitate the use of the prediction values within a reward function. The best model was the LightGBM, and its correlation coefficient for the test dataset was 0.85. The above operations and their results are shown in Fig. S3. Next, a reward function and a configuration were designed via chat interactions based on the following conditions: maximizing the predicted inhibitory activity against EGFR and the QED score; setting the exploration parameter *c* to 0.1; setting the number of generated molecules to 50000; and using Lipinski’s rule-of-five filter and the SAscore filter with a threshold of 4.5. Then, ChemTSv2 was executed via chat using the above reward function and configuration files. Finally, the analysis tool was utilized to analyze the molecule generation results. As shown in the optimization process of Fig. 3 (right panel), ChatChemTS successfully designed molecules with predicted pChEMBL values above seven and QED scores of approximately 0.7. To confirm whether the molecule generation process considering the QED score worked properly, we compared the QED optimization processes of methods generating molecules based on both inhibitory activity and QED scores and methods based only on inhibitory activity under the same conditions. Figure S5 indicates that the molecules designed solely based on inhibitory activity often had QED scores of approximately 0.4, highlighting the effectiveness of incorporating QED scores with inhibitory activity in the reward function.

## Discussion and conclusion

In this study, we introduced ChatChemTS, an LLM-powered application, to facilitate user interactions with ChemTSv2 through an interactive chat interface. Two demonstrations, chromophore and EGFR inhibitor design tasks, were showcased as common de novo molecular generation tasks: single- and multiobjective optimizations, respectively. In the demonstrations, ChatChemTS successfully assisted with four main operations that users need to perform when using AI-based molecule generators: designing reward functions, setting up configurations, executing a molecule generator, and analyzing the results. Despite their success in terms of designing molecules with targeted properties based on prediction models, there is potential for improving the design of the reward functions. For example, the designed EGFR inhibitor lacked the major hinge-binding scaffolds used in common kinase inhibitors [45]. A potential solution to this issue is to introduce a reward function designed to increase the structural similarity to these scaffolds, which is an unsupported feature in ChatChemTS.

The following tasks remain to make the experience of AI-based molecule designs via ChatChemTS more appropriate and convenient. ChatChemTS currently supports a single AI-based molecule generator, ChemTSv2; however, ChatChemTS was designed to easily incorporate additional molecule generators, assuming that the reward design and configuration settings are independently specified in separate files. Similarly, while ChatChemTS can be modified to support seamless switching between multiple LLMs (e.g., Meta’s Llama and Google AI’s Gemini), provided they are supported by LangChain’s Chat Models, this functionality has not yet been implemented. In our experiments, we configured ChatChemTS to retain only the most recent message to avoid saving unintended content during the file-saving step although ChatChemTS can support flexible adjustment of the number of recent messages retained in the GUI setting. Thus, this study did not demonstrate whether the system can work correctly while retaining a long message history. Moreover, the current version of ChatChemTS solely relies on the LLMs, which have learned to use ChemTSv2 through in-context learning techniques, for the quality of the generated reward function and configuration file. In addition to this, considering the inherently probabilistic nature of LLMs, a verification system needs to be developed to ensure that the LLMs correctly interpret user requests and the generated outputs are appropriate. To mitigate the risk of improperly saving reward and configuration files through LLM operations and the associated economic costs, a save button should be implemented within the GUI’s code window. In terms of reward designs, the current reward generator tool is limited to using Python packages and ML-based prediction models within reward functions. Given the frequent use of various simulation packages, such as the Gaussian 16 [46] and AutoDock Vina [47] packages, in material and drug design scenarios, we plan to make these packages available in the reward designs of ChatChemTS. Furthermore, introducing a feature that automatically optimizes reward designs [48] could significantly reduce the manual effort required for reward adjustment, as this process typically involves extensive trial and error. To enhance the user experience in preparing and using ML models, we will add functionality for classification models in ChatChemTS and the Prediction model builder because both are currently designed primarily for regression models. In the configuration setup stage, switching the recurrent neural network (RNN) models provided in ChemTSv2 via ChatChemTS currently requires users to be well-versed in the characteristics of these RNNs. Thus, efforts are underway to improve ChatChemTS so that users can utilize these RNN models without needing such knowledge by enabling appropriate suggestions for selecting the desired RNN model. ChatChemTS currently supports the basic use cases of ChemTSv2, does not yet provide a sufficiently flexible user experience, and would require expert intervention and support for comprehensive AI-based molecule designs. As we continue the development of ChatChemTS, it is crucial to establish a robust software foundation that includes features such as a test suite, continuous integration setup on GitHub, and LLM monitoring capabilities, similar to BioChatter [49]. The current version of ChatChemTS does not have such a robust software foundation, and thus, we also plan to work on building such a foundation.

## Methods

### Large language model

An LLM is a type of AI model that can perform various general-purpose natural language processing (NLP) tasks at the human level, including text generation, question answering, and information extraction [50]. The core architecture of an LLM is a deep learning technique called a transformer [51], and LLMs are generally trained on immense amounts of data. LLMs are frequently utilized in combination with prompting strategies, such as ICL, Chain of Thought (CoT), and Planning, for solving various tasks to enhance their contextual understanding and improve their task-specific performance [50]. In this study, GPT-4 (gpt-4-0613) developed by OpenAI with a temperature of 0.1 and the ReAct framework were used as the LLM and the Planning strategy, respectively.

### ReAct framework

The ReAct framework allows LLMs to intertwine reasoning traces with task-specific actions in external environments, facilitating general task-solving [34]. The LLM that performs the above role is called an agent. Within the ReAct framework, LLMs can interact with external tools, such as Wikipedia, web searches, the Python interactive computing environment, and user-customized tools, and use their feedback for reasoning traces to produce more reliable responses. LangChain was used to implement the ReAct framework in ChatChemTS, and a zero-shot ReAct agent that can return structured outputs to handle the prepared tools in ChatChemTS was used. The system message used in the agent is shown in Listing S1. The following section describes the tools used in ChatChemTS.

### Tools

In the ReAct framework, tools are pivotal for enhancing LLMs by enabling the retrieval of additional information, which contributes to more reliable responses. Notably, the tools included in the proposed application were the minimum tools required for using AI-based molecule generators, but other tools can easily be added to the application to satisfy users’ specific needs. The tools for reward and configuration generations utilized LLMs tuned to the corresponding tasks using few-shot prompting techniques. All the prompts utilized are described in Listing S2 and Listing S3.

#### Reward generator

This tool is dedicated to designing reward functions in ChemTSv2 format and is based on an LLM. A few-shot prompting technique was used to steer the LLM to the reward design tool. All the utilized prompts are described in Listing S2. Upon receiving a user request for molecules optimized based on specific properties, this tool returns a reward function reflecting that request. The currently available molecular properties are those that can be calculated by RDKit software and predicted using prediction models obtained from the FLAML [52]. There is no limit to the number of properties that can be used for multiobjecitve optimization in ChemTSv2, though the success of the optimization task is a separate issue. It should be noted that we do not explicitly teach the LLM how to calculate specific molecular properties through the prompts, so the ability to code a program that accurately computes these properties depends on the LLMs’ performance.

#### Prediction model builder

This tool offers a GUI application that allows users to build their own prediction models using an automated ML tool (the FLAML) [52]. The interface was designed to be accessible to users with varying levels of ML expertise. The application accepts two input types: a CSV file for building general prediction models and a UniProt [53] ID for constructing quantitative structure-activity relationship (QSAR) models from the ChEMBL database.

When a user uploads a CSV file, it is rendered as a table on the interface to enable the user to verify its content. To build prediction models, the user must select the names of two columns: one containing molecule structures in the SMILES format and another containing a target variable. A Morgan fingerprint, with a radius of two and dimensions of 2048, is used as an input feature for the prediction models. The interface guides the user through a structured configuration process to develop a prediction model with the FLAML. Initially, the user adjusts the data proportion for the test dataset. Following this, the user has the option to select the “use auto” for default ML estimators or manually choose ML estimators from a list option, including a random forest, LightGBM, eXtreme Gradient Boosting (XGBoost), Categorical Boosting (CatBoost), Extremely Randomized Trees (Extra Trees), Logistic Regression with L1 or L2 regularization, and k-nearest neighbors. In the FLAML, the list of the default estimators are defined as follows: LightGBM, random forest, XGBoost, CatBoost, Extra Trees. Then, the user specifies the type of ML task to perform, which is currently limited to regression, and the metric used to evaluate the performance of the constructed model during training, with the option to utilize automatic metric selection. If the automatic metric selection is chosen, the FLAML uses $1-r^2$ as the minimizing error metric, where $r^2$ is the coefficient of determination. A time budget can be set to manage the amount of time that computational resources are dedicated to performing the AutoML search process. If the values of the target variable are not normalized, the user should employ the standardization function in this application to standardize the target values, ensuring that reward functions can be appropriately designed. Once all the settings are finalized, this tool runs the AutoML process to find quality models and saves the model that performs best on the test dataset for use in a reward function.

When a user inputs a UniProt ID, the application fetches and processes data from the ChEMBL database using the ChEMBL webresource client package [54]. The application verifies the existence of records for the specified UniProt ID and then allows users to refine the dataset by deduplicating molecules based on pChEMBL values, retaining records according to their specific assay types, and filtering out records with certain assay descriptions and activity types. The retrieved record items that can be checked by users are as follows: canonical SMILES string; pChEMBL value; assay description, ChEMBL ID, and type; standard type, value, units, and relation; molecule ChEMBL ID; organism and ChEMBL ID related to the input UniProt ID; and document ChEMBL ID. Subsequently, the workflow is the same as that of the process described above after uploading a CSV file. A demonstration movie of this tool is shown in Supplementary Movie 2.

#### Configuration generator

This tool is specialized in generating configuration files in YAML format for ChemTSv2 and is based on an LLM. The configuration includes, for example, the number of generated molecules, the exploration parameter *c*, and molecular filters for skipping the reward calculation (refer to the paper that presented ChemTSv2 for details [11]). Similar to the reward generator tool, this tool employs a few-shot prompting technique to generate configurations. All the prompts used are described in Listing S3. By taking a user request for setting a ChemTSv2 configuration, this tool returns a configuration reflecting that request.

#### ChemTSv2 API

This tool serves as an API for executing ChemTSv2. ChemTSv2 employs two primary algorithms: a recurrent neural network (RNN) to generate molecules and a Monte Carlo Tree Search (MCTS) to navigate the search space. The usage workflow of ChemTSv2 involves four steps: preparing reward and configuration files, executing ChemTSv2, and analyzing the generated molecules, all of which are supported through chat-based interactions in ChatChemTS. When using ChemTSv2 directly, there are no software restrictions for designing reward functions, but the software users want to use must be installed in the computational environment beforehand. Currently, ChemTSv2 can only use pre-installed software, RDKit and FLAML, for reward design, as described in the Reward generator section. As for the API tool, upon receiving a path to a configuration file, the tool runs a ChemTSv2 using the provided configuration and returns a path to the execution outcome. The API was built using the FastAPI package [39].

#### Molecule generation analyzer

This tool provides users with a GUI application to easily analyze the results of molecule generation processes. Once a user uploads a CSV result file, the application offers three interactive functions, a table viewer, a molecule viewer, and a time series viewer, which are the common features used to analyze molecule generation results. The GUI application was developed based on Streamlit [40] for creating the user interface, pandas [55] for manipulating molecule generation results, and mols2grid [56] for interactively visualizing molecules. A demonstration movie of this tool is shown in Supplementary Movie 3.

## Availability and requirements

- Project name: ChatChemTS
- Project home page: https://github.com/molecule-generator-collection/ChatChemTS
- Operating system(s): Tested on Linux OS (Ubuntu 22.04.2 LTS), macOS (Ventura 13.6.8 and Sonoma 14.4.1), and Windows OS with the Windows Subsystem for Linux (11 Pro 23H2, 11 Home 23H2, 10 Pro 22H2).
- Programming language: Python 3.
- Other requirements: dependencies are described in the README file on the project home page.
- License: MIT
- Any restrictions to use by non-academics: none.

**Supporting information available**

Snapshot UI of prediction model builder tool when building the prediction models to predict absorption wavelength (Fig. S1); Snapshot UI of prediction model builder tool when building the prediction model to predict inhibitory activity against EGFR (Fig. S2); Comparison of QED optimization processes between generating molecules considering inhibitory activity against EGFR and QED score and generating molecules solely considering the inhibitory activity (Fig. S3); System message used in the agent of ChatChemTS (Listing S1); Few-shot prompting for the reward generator tool (Listing S2); Few-shot prompting for config generator tool (Listing S3); Demonstration movies for ChatChemTS example workflow (Supplementary Movie 1), prediction model builder tool (Supplementary Movie 2), and molecule generation analyzer tool (Supplementary Movie 3).

## Supplementary Information

Supplementary Maretial 1.

Supplementary Maretial 2.

Supplementary Maretial 3.

Supplementary Maretial 4.

## Data Availability

The ChatChemTS application is publicly available on GitHub at https://github.com/molecule-generator-collection/ChatChemTS under the MIT License. The README file in the GitHub repository provides information about how to set up and use the application. The tutorials on ChatChemTS are available on GitHub at https://github.com/molecule-generator-collection/ChatChemTS/wiki/Tutorial.
