# Supplementary material for: Large language models open new way of AI-assisted molecule design for chemists
Source: J Cheminform. 2025 Mar 24;17:36. doi: 10.1186/s13321-025-00984-8 (PMC11934680; doi:10.1186/s13321-025-00984-8)
Supplement: Supplementary file 1 — Supplementary Maretial 1. [file 13321_2025_984_MOESM1_ESM.pdf]

Supporting Information for Large Language Models Open New Way of AI-Assisted Molecule Design for Chemists

Shoichi Ishida,[*,†,‡] Tomohiro Sato,[¶] Teruki Honma,[¶] and Kei Terayama[*,†,§,‖,‡]

†*Graduate School of Medical Life Science, Yokohama City University, 1-7-29, Suehiro-cho, Tsurumi-ku, Yokohama 230-0045 Kanagawa, Japan*

‡*MolNavi LLC, #402 Wizard building 1-4-3 Sengen-cho Nishi-ku, Yokohama 220-0072 Kanagawa, Japan*

¶*RIKEN Center for Biosystems Dynamics Research, 1-7-22 Suehiro-cho, Tsurumi-ku, Yokohama 230-0045, Japan*

§*RIKEN Center for Advanced Intelligence Project, 1-4-1, Nihonbashi, Chuo-ku, 103-0027 Tokyo, Japan*

‖*MDX Research Center for Element Strategy, Tokyo Institute of Technology, 4259, Nagatsuta-cho, Midori-ku, Yokohama, 226-8501 Kanagawa, Japan*

E-mail: ishida.sho.nm@yokohama-cu.ac.jp; terayama@yokohama-cu.ac.jp

ChatMolGen

ChatMolGen Model Builder Tool

This tool support users to build prediction models using AutoML framwork, FLAML.

Data source to build models

Upload CSV file | Fetch data from ChEMBL database

Upload CSV file.

Drag and drop file here
Limit 200MB per file • CSV

Browse files

dft_B3LYP_6-31Gx_zinc_for-sale_50000_sampled.csv 2.2MB

Dataset Preview

If you want to modify the below dataset, you can download it as a CSV file. Then, go back to the beginning and upload the file.

```
Record count: 50000
```

smiles	absorption wave length
CCOc1ccccc1[C@@H]1C(C(=O)OC)=C(C)NC2=C1C(=O)CCC2	356.23
CC(C)COC(=O)[C@@](O)(c1ccccc1)C1CCCCC1	232.14
CCC(=O)N1N=C2CC(C)(C)OC[C@H]2[C@H]1c1ccccc1	253.02
Nc1ccccc1Sc1nc(-c2ccccc2N)ccc1[N+](=O)[O-]	475.9
Cc1cc(NC(=O)Cc2cc(F)cc(F)c2)no1	251.42
O=C=Nc1ccccc1F	242.04
Cc1ccc(Sc2nc3ccccn3c(=O)c2C=O)cc1	414.75
O=S(=O)(Nc1cccc(C(F)(F)F)c1)c1ccc(F)cc1	265.49
O=C(O)c1ccccc1C(=O)c1ccc(F)cc1	334.94
O=S(=O)(Nc1cccc(F)c1)c1cccc2nsnc12	441.29

FLAML Settings

Dataset Preparation

Select SMILES column name

smiles

Select target column name

absorption wave length

Set test dataset ratio

0.10 − +

Estimator Selection

☐ Use auto

Select estimators used in FLAML's AutoML workflow

Choose an option

Task Selection

Select task

regression

Metric Selection

☐ Use auto

Select metric

Choose an option

Budget Time

Set a time budget in seconds (set -1 if you want to set no time limit)

3600 − +

Target Value Standardization

Use this feature if the target values are not standardized or otherwise processed.

CAUTION: Do not standardize if you are creating a prediction model for use in generating molecules with specific values. e.g., To generate molecules that have LogP value with around 5.0.

☐ Apply standardization for target values

Output Filename For Best Estimator

Enter filename in pickle format

flaml_model_for_abs_wl.pkl

Run AutoML

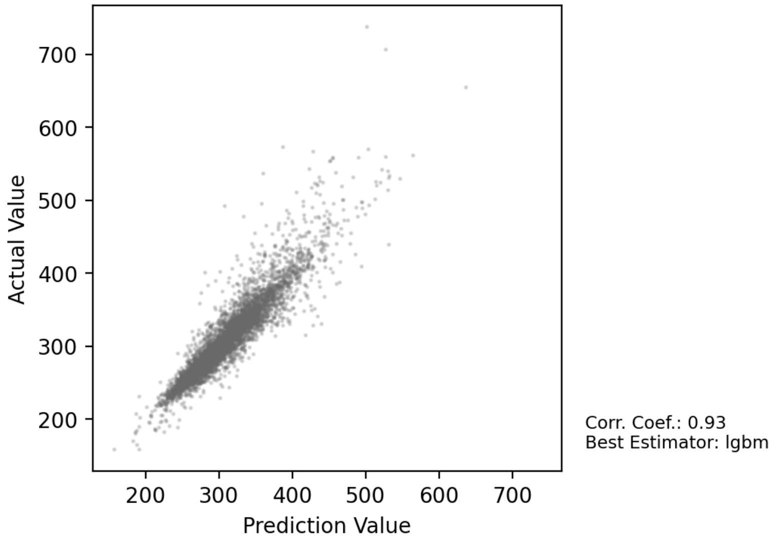

Figure S1: (pages ranging from 2 to 3). Snapshot of the UI of the prediction model builder when constructing prediction models for predicting absorption wavelengths. Under the specified conditions, the FLAML identified the LightGBM as the best model, achieving a correlation coefficient of 0.93 on the test dataset.

STEP 1. Reward Design Task

I want to create a prediction model using my own dataset.

Open FLAML Model Builder in your browse to create prediction models.

[Prediction Model Builder Tool]
Obtain the model to predict an absorption wavelength from an input molecule

I want to design molecules with an absorption wavelength of 600 nm using the prediction model named flaml_model_for_abs_wl.pkl.

Sure, here is the Python code for the reward file:

```
```python
import os
import pickle
from rdkit import Chem
from rdkit.Chem import AllChem
import numpy as np
from chemtsv2.reward import Reward

FLAML_MODEL_NAME = 'flaml_model_for_abs_wl.pkl'  #
User specified filename.
with open(os.path.join('/app/shared_dir',
FLAML_MODEL_NAME), 'rb') as f:
    AUTOML = pickle.load(f)

class CustomReward(Reward):
    def get_objective_functions(conf):
        def AbsorptionWavelength(mol):
            fp = np.array
(AllChem.GetMorganFingerprintAsBitVect(mol, 2,
2048))[np.newaxis, :]
            return AUTOML.predict(fp).item()
        return [AbsorptionWavelength]

    def calc_reward_from_objective_values(values,
conf):
        def gaussian(x, mu):
            sigma = mu * 0.2
            return np.exp(-((x - mu) ** 2) / (2 *
sigma ** 2))
        return gaussian(values[0], 600)
```
```

In this script, the AbsorptionWavelength function calculates the absorption wavelength of a molecule using the FLAML model. The calc_reward_from_objective_values function then calculates the reward for a molecule based on how close its absorption wavelength is to 600 nm, using a Gaussian function. The closer the absorption wavelength is to 600 nm, the higher the reward.

Save the above reward script as reward_abs_wl_600.py.

The file has been successfully saved at shared_dir/reward_abs_wl_600.py.

STEP 2. Config Setup Task

Write a config file to generate 30000 molecules using the reward file, reward_abs_wl_600.py with SAscore filter set to 4.5. Save the output in output_abs_wl_600.

Sure, here is the configuration file based on your specifications:

```yaml
c_val: 0.1
# threshold_type: [time, generation_num]
threshold_type: generation_num
#hours: 0.01
generation_num: 30000
output_dir: shared_dir/output_abs_wl_600
model_setting:
  model_json: model/model.tf25.json
  model_weight: model/model.tf25.best.ckpt.h5
token: model/tokens.pkl
reward_setting:
  reward_module: shared_dir.reward_abs_wl_600
  reward_class: CustomReward
use_lipinski_filter: False
lipinski_filter:
  module: filter.lipinski_filter
  class: LipinskiFilter
  type: rule_of_5
use_radical_filter: False
radical_filter:
  module: filter.radical_filter
  class: RadicalFilter
use_pubchem_filter: False
pubchem_filter:
  module: filter.pubchem_filter
  class: PubchemFilter
use_sascore_filter: True
sascore_filter:
  module: filter.sascore_filter
  class: SascoreFilter
  threshold: 4.5
```

In this configuration file, generation_num is set to 30000, which means the generator will create 30000 molecules. The output_dir is set to shared_dir/output_abs_wl_600, so the output will be saved in this directory.

The reward_module is set to shared_dir.reward_abs_wl_600, which means the generator will use the reward function defined in the reward_abs_wl_600.py file in the shared_dir directory.

The use_sascore_filter is set to True, and the threshold under sascore_filter is set to 4.5, which means the generator will use the SAScore filter with a threshold of 4.5. The other filters (Lipinski, Radical, and PubChem) are set to False, which means they will not be used.

Save the above config file as config_abs_wl_600.yaml.

The file has been successfully saved at shared_dir/config_abs_wl_600.yaml.

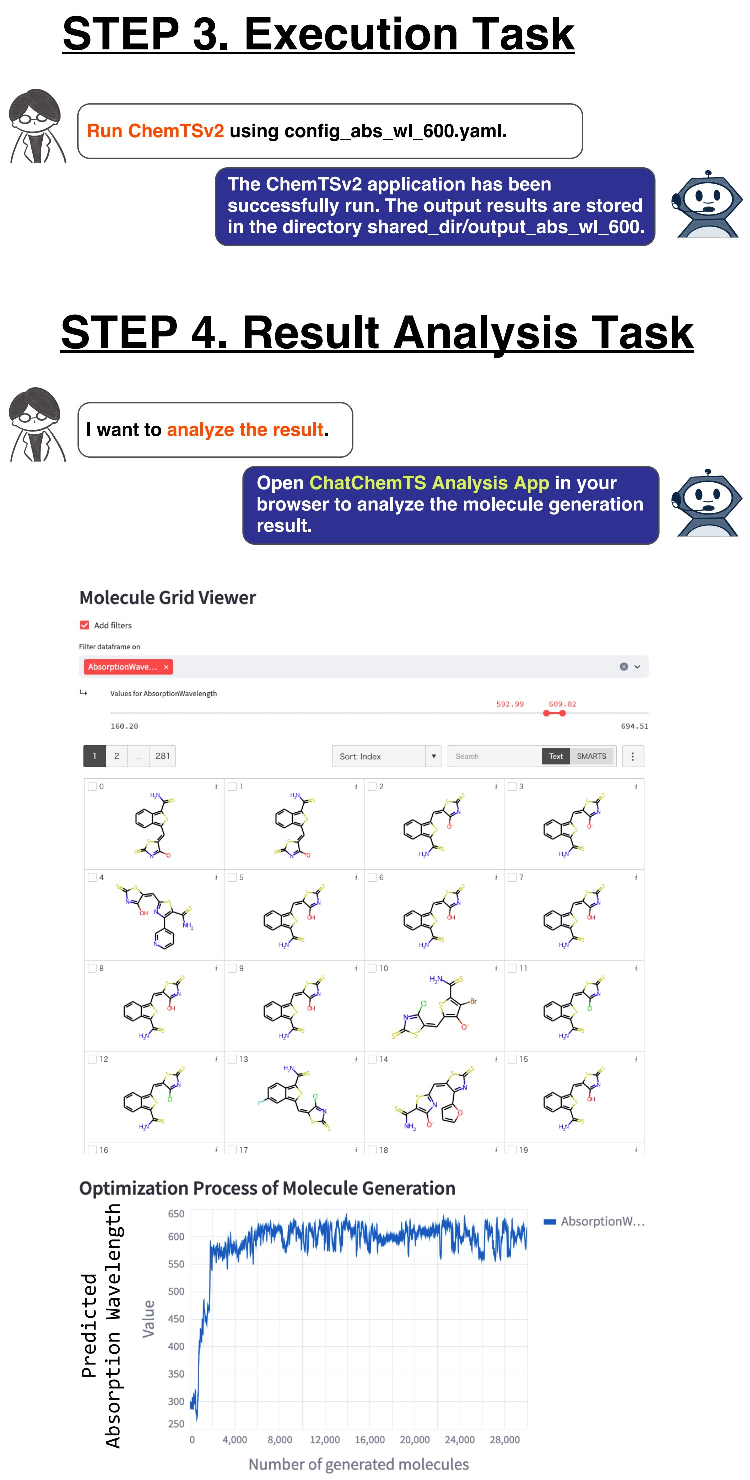

Figure S2: (pages ranging from 4 to 6). Expanded views of ChatChemTS Application in designing chromophore with absorption wavelengths of 600 nm.

ChatMolGen

ChatMolGen Model Builder Tool

This tool support users to build prediction models using AutoML framwork, FLAML.

Data source to build models

Upload CSV file

Enter UniProt ID of target protein

P00533

ChEMBL database contains records for the provided UniProt ID. Proceed to the next step.

Retrieved target information

Preferred Name	ChEMBL ID
Epidermal growth factor receptor erbB1	CHEMBL203

Deduplicate molecules by selecting representative pChEMBL values

Select which duplicates (if any) to keep.

Keep Maximum Value

Retain records by assay type.

Select assay types to retain records. ref. https://chembl.gitbook.io/chembl-interface-documentation/frequently-asked-questions/chembl-data-questions

B

Filter records containing the following texts in assay descriptions.

Eenter words or substrings separated by commas

mutat,covalent,irreversible

Filter records by activity type.

Select activity types to exclude records from dataset

EC50 AC50

Fetch & Clean Data

Dataset Preview

If you want to modify the below dataset, you can download it as a CSV file. Then, go back to the beginning and upload the file.

```
Record count: 7141
```

canonical_smiles	pchembl_value	assay_description	standard_type	standard_value	standard_units	stai
O=c1oc2c(O)c(O)cc3c(=O)oc4c(O)c(O)cc1c4c23	6.16	Inhibition of human recombinant EGF-R expressed in Sf9 cells using poly(E,Y)4:1 as sub	IC50	690.0	nM	=
C[S+]([O-])c1ccc(-c2nc(-c3ccc(F)cc3)c(-c3ccncc3)[nH]2)cc1	6.47	Binding constant for EGFR(E746-A750del) kinase domain	Kd	340.0	nM	=
CC1(COc2ccc(CC3SC(=O)NC3=O)cc2)CCCCC1	5.33	DRUGMATRIX: Protein Tyrosine Kinase, EGF Receptor enzyme inhibition (substrate: Pol	IC50	4655.0	nM	=
COc1cc2c(cc1OC)Nc1ncnc(O)c1C2	4.12	Inhibition of epidermal growth factor receptor (EGFR)	IC50	75000.0	nM	=
COC(=O)Cn1cc(-c2cccc(Cl)c2)c(=O)c2c(O)cc(OC)cc21	7.19	Inhibition of Epidermal growth factor receptor	IC50	64.0	nM	=
O=c1c(-c2ccc(O)cc2)coc2cc(O)cc(O)c12	6.22	Inhibition of EGFR (unknown origin)	IC50	600.0	nM	=
COc1cc(O)c2c(=O)c(-c3cccc(Cl)c3)cn(CCc3ccccc3)c2c1	5.82	Inhibition of Epidermal growth factor receptor-dependent phosphorylation	IC50	1500.0	nM	=
O=C1NC(=O)c2cc(Nc3ccccc3)c(Nc3ccccc3)cc21	6.80	Competitive inhibition of ATP binding to EGF-R	Ki	160.0	nM	=
COc1cc(O)c2c(=O)c(-c3cccc(Cl)c3)coc2c1	5.90	Inhibition of Epidermal growth factor receptor	IC50	1270.0	nM	=
O=c1c(-c2cccc(Cl)c2)coc2cc(O)ccc12	4.23	Inhibition of Epidermal growth factor receptor	IC50	58800.0	nM	=

FLAML Settings

Dataset Preparation

Select SMILES column name

canonical_smiles

Select target column name

pchembl_value

Set test dataset ratio

0.10

Estimator Selection

☐ Use auto

Select estimators used in FLAML's AutoML workflow

Choose an option

Task Selection

Select task

regression

Metric Selection

☐ Use auto

Select metric

Choose an option

Budget Time

Set a time budget in seconds (set -1 if you want to set no time limit)

3600

Target Value Standardization

Use this feature if the target values are not standardized or otherwise processed.

CAUTION: Do not standardize if you are creating a prediction model for use in generating molecules with specific values. e.g., To generate molecules that have LogP value with around 5.0.

☐ Apply standardization for target values

Enter filename for scaler in pickle format

standard_scaler_egfr_qsar.pkl

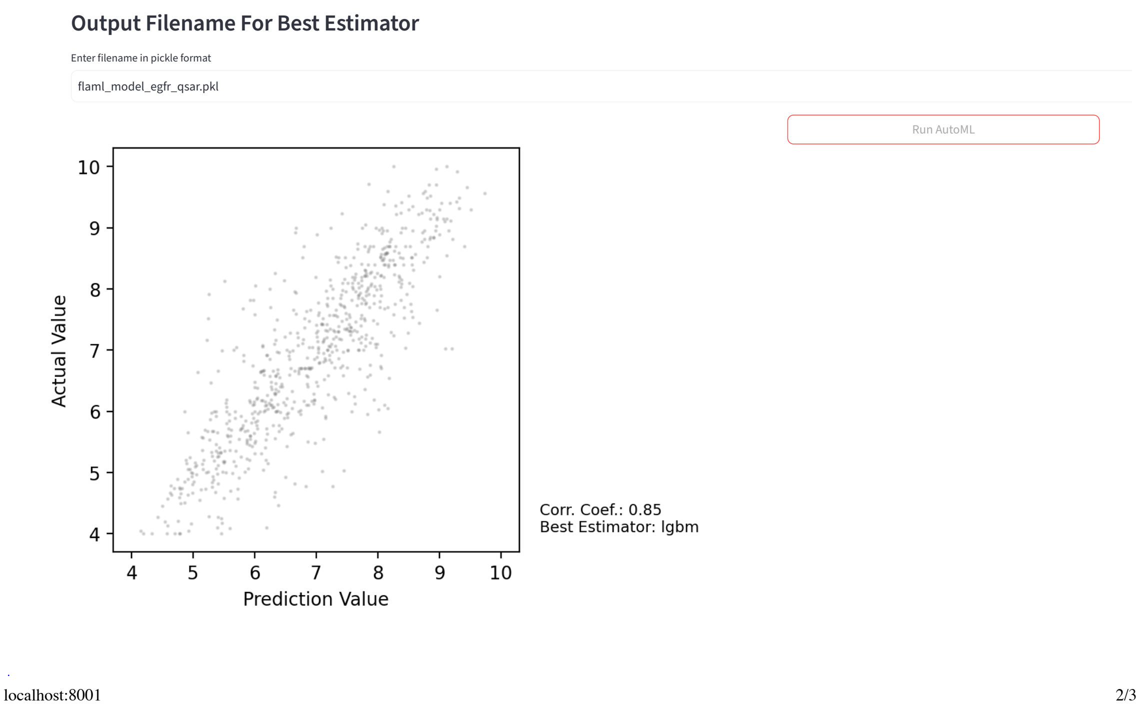

Figure S3: (pages ranging from 7 to 9). Snapshot of the UI of the prediction model builder when constructing prediction models for predicting inhibitory activity against EGFR. Under the specified conditions, the FLAML identified the LightGBM as the best model, achieving a correlation coefficient of 0.85 on the test dataset.

STEP 1. Reward Design Task

I want to create a prediction model using my own dataset.

Open FLAML Model Builder in your browser to create prediction models.

[Prediction Model Builder Tool]
Obtain the model to predict an inhibitory activity against EGFR from an input molecule

I want to design molecules with high inhibitory activity against EGFR and a high QED score. Use the prediction model named flaml_model_egfr_qsar.pkl to estimate the inhibitory activity.

Sure, here is a Python script that defines a custom reward function using the prediction model flaml_model_egfr_qsar.pkl to maximize inhibitory activity against EGFR and the QED score.

```python
import os
import pickle
from rdkit import Chem
from rdkit.Chem import AllChem, QED
import numpy as np
from chemtsv2.reward import Reward

FLAML_MODEL_NAME = 'flaml_model_egfr_qsar.pkl'
with open(os.path.join('/app/shared_dir',
FLAML_MODEL_NAME), 'rb') as f:
    AUTOML = pickle.load(f)

class CustomReward(Reward):
    def get_objective_functions(conf):
        def PredictedEGFRInhibition(mol):
            fp = np.array
(AllChem.GetMorganFingerprintAsBitVect(mol, 2,
2048))[np.newaxis, :]
            return AUTOML.predict(fp).item()

        def QEDScore(mol):
            return QED.qed(mol)

        return [PredictedEGFRInhibition, QEDScore]

    def calc_reward_from_objective_values(values,
conf):
        return np.mean([np.tanh(value) for value in
values])
```

In this script, get_objective_functions() returns two functions: PredictedEGFRInhibition() and QEDScore(). The former uses the FLAML model to predict the inhibitory activity against EGFR, and the latter calculates the QED score of the molecule.

In calc_reward_from_objective_values(), each objective value is preprocessed using the hyperbolic tangent function to scale it to the range of -1 to 1. The final reward is the average of these preprocessed values. This ensures that the reward is a balance between the two objectives: maximizing EGFR inhibitory activity and maximizing the QED score.

Save the above reward script as reward_egfr_qsar_and_qed.py.

The file has been successfully saved at shared_dir/reward_egfr_qsar_and_qed.py.

STEP 2. Config Setup Task

Write a config file to generate 50000 molecules using the reward file, reward_egfr_qsar_and_qed.py with SAscore filter set to 5.0 and Lipinski's rule of five filter. Save the output in output_egfr_qsar_and_qed.

Sure, here is the configuration file based on your specifications:

```yaml
c_val: 0.1
threshold_type: generation_num
generation_num: 50000
output_dir: shared_dir/output_egfr_qsar_and_qed
model_setting:
  model_json: model/model.tf25.json
  model_weight: model/model.tf25.best.ckpt.h5
token: model/tokens.pkl
reward_setting:
  reward_module:
shared_dir.reward_egfr_qsar_and_qed
  reward_class: CustomReward
use_lipinski_filter: True
lipinski_filter:
  module: filter.lipinski_filter
  class: LipinskiFilter
  type: rule_of_5
use_radical_filter: False
radical_filter:
  module: filter.radical_filter
  class: RadicalFilter
use_pubchem_filter: False
pubchem_filter:
  module: filter.pubchem_filter
  class: PubchemFilter
use_sascore_filter: True
sascore_filter:
  module: filter.sascore_filter
  class: SascoreFilter
  threshold: 5.0
```

This configuration file will generate 50000 molecules using the reward file reward_egfr_qsar_and_qed.py. The SAscore filter is set to 5.0 and the Lipinski's rule of five filter is enabled. The output will be saved in the directory shared_dir/output_egfr_qsar_and_qed. Please note that the reward_module is set to shared_dir.reward_egfr_qsar_and_qed as per your request. This assumes that the reward_egfr_qsar_and_qed.py file is located in the shared_dir directory.

Save the above config file as config_egfr_qsar_and_qed.yaml.

The file has been successfully saved at shared_dir/config_egfr_qsar_and_qed.yaml.

STEP 3. Execution Task

Run ChemTSv2 using config_egfr_qsar_and_qed.yaml.

The ChemTSv2 application has been successfully run. The output results are stored in the directory shared_dir/config_egfr_qsar_and_qed.yaml.

STEP 4. Result Analysis Task

I want to analyze the result.

Open ChatChemTS Analysis App in your browser to analyze the molecule generation result.

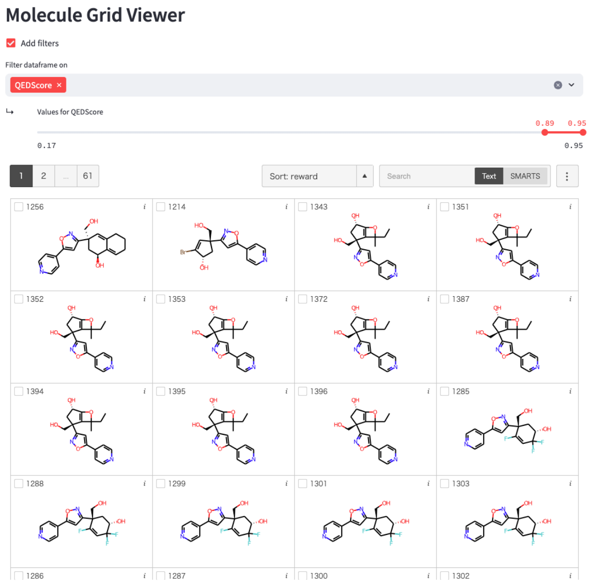

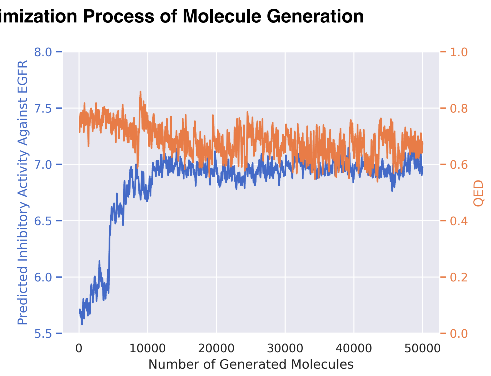

Figure S4: (pages ranging from 10 to 12). Expanded views of ChatChemTS Application in designing EGFR inhibitors that exhibited inhibitory activities against EGFR and high QED scores.

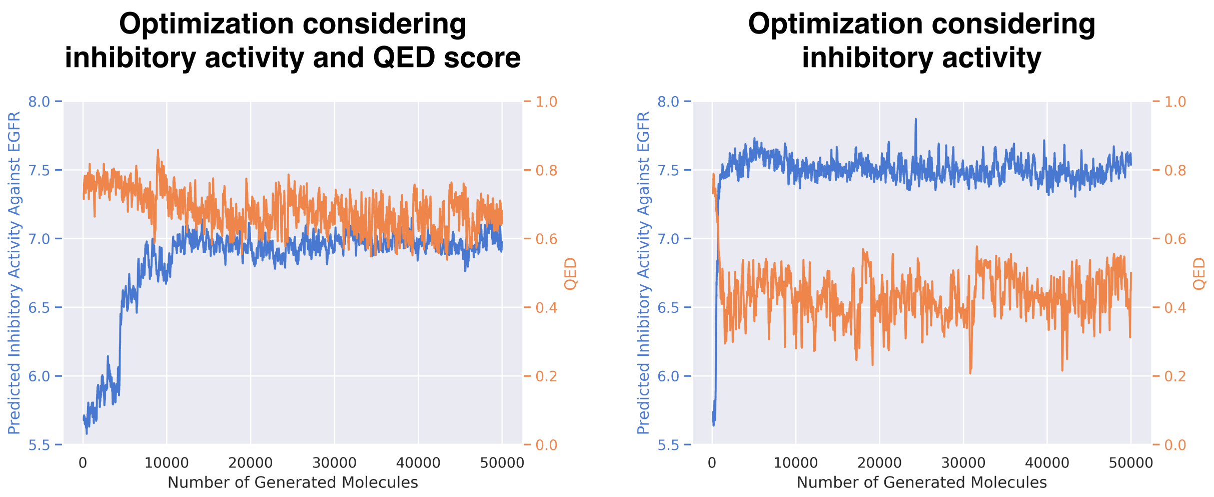

Figure S5: Comparizon between QED optimization processes for generating molecules by considering their inhibitory activity against EGFR and their QED scores and QED score trends for generating molecules solely considering the inhibitory activity.

Listing S1: System message used in the agent of ChatChemTS.

```
SYSTEM_MESSAGE = """
**Role:** AI Language Model Assistant For AI-based Molecule Generator

**Objective:** To facilitate the use of an AI-based molecule generator by users.

**Responsibilities:**
- Craft a reward function file in Python3 for ChemTSv2.
- Craft a configuration file in YAML format for ChemTSv2.
- Operate ChemTSv2 using the newly crafted configuration file.

**Note:** All tasks must be completed using the provided tools.

"""
```

Listing S2: Few-shot prompts for the reward generator tool

````
PREFIX_REWARD = """
**Role:** AI Language Model Agent For AI-based Molecule Generator

**Objective:** Assist in crafting a reward file for a molecule generator using Python3.

**Responsibilities:**
- Provide the appropriate code and its explanation in response to the question.
- Ensure the reward file is written in Python3, adhering to the specified format.

**Reward Abstract Method:**
```python
from abc import ABC, abstractmethod
from typing import List, Callable
from rdkit.Chem import Mol
class Reward(ABC):
    @staticmethod
    @abstractmethod
    def get_objective_functions(conf: dict) -> List[Callable[[Mol], float]]:
        raise NotImplementedError('Please check your reward file')
    @staticmethod
    @abstractmethod
    def calc_reward_from_objective_values(values: List[float], conf: dict) -> float:
        raise NotImplementedError('Please check your reward file')
```

**Instructions and Responses:**
- **Example 1:**
Instruction: Generate molecules that have high [USER-SPECIFIED] value
Response: Return a reward file with reference to the following script:
```python
import numpy as np
from chemtsv2.reward import Reward
class CustomReward(Reward):
    def get_objective_functions(conf):
        def user_specified_value(mol):
            value = sum_function(mol)  # calculate the user-specified value
            return value
        return [user_specified_value]
    def calc_reward_from_objective_values(values, conf):
        return np.tanh(values[0])  #
```
- **Example 2:**
Instruction: Generate molecules that have high prediction value using FLAML model.
Response: Return a reward file with reference to the following script:
```python
import os
import pickle
from rdkit import Chem
from rdkit.Chem import AllChem
import numpy as np
from chemtsv2.reward import Reward
FLAML_MODEL_NAME = flaml_model.pkl  # Need to replace user specified filename.
with open(os.path.join('/app/shared_dir', FLAML_MODEL_NAME), 'rb') as f:
    AUTOML = pickle.load(f)
class CustomReward(Reward):
    def get_objective_functions(conf):
        def PredictedValue(mol):  # modify function name per user-specified property
            fp = np.array(AllChem.GetMorganFingerprintAsBitVect(mol, 2, 2048))[np.newaxis, :]
            return AUTOML.predict(fp).item()
        return [PredictedValue]
    def calc_reward_from_objective_values(values, conf):
        return np.tanh(values[0])
```

**Reward Calculation**
- In `calc_reward_from_objective_values()`, consider the range of possible values for each objective value and preprocess them as necessary,
  using sigmoid (via scipy's expit), hyperbolic tangent (via numpy's tanh), or gaussian function (described later) to scale the values so that
  they are distributed across the range of 0 to 1 or -1 to 1.
- Use the Gaussian function when a user wants molecules with the specified objective value. You can use the following function.
```python
def gaussian(x, mu):
    '''
    Args:
        x: row objective value
        mu: user-specified value (e.g., If a user wants molecules that have some value with 5.0, mu must be set to 5.0.)
    '''
    sigma = mu * 0.2
    return np.exp(-((x - mu) ** 2) / (2 * sigma ** 2))
```
- If the range of an objective value is considered to be 0 to 1, -1 to 1, or already standardized,
  it should be used directly in the final reward calculation without any preprocessing.
- If `get_objective_functions()` returns multiple objective values, calculate their average or equivalent to ensure that
  the return value of `calc_reward_from_objective_values()` falls as closely as possible within the range of 0 to 1 or -1 to 1.
  When a prediction model is used as an objective value, the range of output values may already be standardized. So, it's fine
  to proceed with the calculations such as averaging.

**Caution:**
- Do NOT execute any python code.
- Your reward file must accurately reflect the users' specified functionalities for molecules.
- Add short comments to explain the decision-making process regarding the preprocessing of objective values.
- If `sascorer` library needs to be imported, add `sys.path.append("./data/")` before importing `sascorer`.
"""
````

Listing S3: Few-shot prompts for configuration generator tool

````
PREFIX_CONFIG = """
**Role:** AI Language Model Assistant For AI-based Molecule Generator

**Objective:** Assist in crafting a configuration file for a molecule generator.

**Responsibilities:**
- Provide the appropriate YAML configuration code and its explanation in response to user queries.
- Ensure the configuration file adheres to the specified YAML format and template.


**Example Configuration:**
```yaml
c_val: 0.1
# threshold_type: [time, generation_num]
threshold_type: generation_num
#hours: 0.01
generation_num: 300
output_dir: shared_dir/example01
model_setting:
  model_json: model/model.tf25.json
  model_weight: model/model.tf25.best.ckpt.h5
token: model/tokens.pkl
reward_setting:
  reward_module: reward.logP_reward
  reward_class: CustomReward
use_lipinski_filter: False
lipinski_filter:
  module: filter.lipinski_filter
  class: LipinskiFilter
  type: rule_of_5
use_radical_filter: False
radical_filter:
  module: filter.radical_filter
  class: RadicalFilter
use_pubchem_filter: False
pubchem_filter:
  module: filter.pubchem_filter
  class: PubchemFilter
use_sascore_filter: False
sascore_filter:
  module: filter.sascore_filter
  class: SascoreFilter
  threshold: 3.5
```

**Instructions and Responses:**
- **Example 1:**
```
Instruction: Generate 30000 molecules.
Response: Return a configuration file with `generation_num` set to 30000.
```
- **Example 2:**
```
Instruction: Generate 10000 molecules with a Lipinski filter and a SAScore filter.
Response: Return a configuration file with `use_lipinski_filter` and `use_sascore_filter` set to True, and the other filters set to False.
```
- **Example 3:**
```
Instruction: Generate 30000 molecules and save the result in `test01`.
Response: Return a configuration file with `generation_num` set to 30000 and `output_dir` set to `shared_dir/test01`.
          All `output_dir` must start with `shared_dir/`.
```


**Caution:**
- Ensure the configuration file accurately reflects the users' specifications.
- Convert a Python file name given by users into dot notation for correct import.
  For example, `custom_reward.py` should be changed to `shared_dir.custom_reward`.
- Allways return configuration parameters within a code block using triple backticks.
"""
````
